# “There’s Not Really Much Consideration Given to the Effect of the Climate on NCDs”—Exploration of Knowledge and Attitudes of Health Professionals on a Climate Change-NCD Connection in Barbados

**DOI:** 10.3390/ijerph17010198

**Published:** 2019-12-27

**Authors:** Roxanne A. Springer, Susan J. Elliott

**Affiliations:** Department of Geography and Environmental Management, University of Waterloo, Waterloo, ON N2L 3G1, Canada; rspringe@uwaterloo.ca

**Keywords:** climate change, non-communicable diseases, behavioral risk factors, vulnerability assessment

## Abstract

Despite widespread awareness of the rise of non-communicable diseases (NCDs) and the growing threat of climate change, little research has explored future health outcomes that will occur at the intersection of these challenges. Ten Barbadian health professionals were interviewed to assess their knowledge of health risks of climate change as it relates to NCDs in Barbados as a case study of a small island state at risk. There is widespread concern among health professionals about the current and future prevalence of non-communicable diseases among Barbadians. There is less concern about the future burden of NCDs in the context of a changing climate, largely because of a lack of knowledge among the majority of the health experts interviewed. Those knowledgeable about potential connections noted the difficulty that climate change would pose to the prevention and management of NCDs, given the impacts of climate stressors to food security, the built environment, and physiological and psychosocial health impacts. Lack of awareness among health professionals of the risk climate change poses to NCD prevalence and impact is reflective of the country’s health priorities that fail to recognize the risk of climate change. We recommend efforts to disseminate information about climate change to stakeholders in the health sector to increase awareness.

## 1. Introduction

Humans are living in a world of unprecedented environmental, technological and social changes, occurring at rapid spatial and temporal scales. These changes contribute to some of the great global health challenges the world is facing. Among these challenges, ‘air pollution & climate change’ and non-communicable diseases (NCDs) are considered to be two of greatest threats to human health and wellbeing [1]. Barbados, like many other small island developing states (SIDS) in the Caribbean, faces the dual burden of climate change impacts and an increasing prevalence of persons living with non-communicable diseases. This article adds to the small body of the literature on health impacts of climate change in the Caribbean, specifically focusing on potential connections between NCDs and climate change.

Decades of research have established that anthropogenic climate change—linked to widespread human activity over the past five decades—poses a risk to natural and human systems [2,3]. One area of marked concern is the effect climatic changes will have on human health and wellbeing. As noted by the 2009 Lancet Commission, climate change is “the greatest threat to global health of the 21st century” [4]. Several key publications have been instrumental in bringing attention to the range of direct and indirect health and wellbeing impacts of climate change [4,5,6,7]. Impacts include loss of life and injuries; heat related illnesses; a proliferation of food-, water- and vector-borne diseases; food insecurity and undernutrition; loss of livelihoods and cultural identities; and forced migration [4,5,6,7]. The health impacts of climate change are associated with climate stressors like rising temperatures, changing precipitation patterns, and increases in the frequency and intensity of extreme weather events [6]. Projections from the World Health Organization (WHO) hold that between 2030 and 2050, there will be approximately 250,000 additional deaths caused by climate change, resulting in between USD two to four billion dollars in direct cost to health a year by 2030 [8]. At risk of loss are the strides made in development and global health over the past fifty years [4].

Even as health systems begin to grapple with the effects of climate change, non-communicable diseases are an on-going burden to population health around the world in both developed and developing countries. According to the WHO, the burden of NCDs has reached epidemic proportions and are the leading cause of deaths worldwide [9]. In 2016, NCDs were responsible for 41 million deaths—71% of all global deaths—namely heart diseases, strokes, cancers, type 2 diabetes and chronic respiratory illnesses [9]. While it was previously thought that the burden of NCDs was concentrated in high-income countries, current data show that the burden is greatest in low-middle income countries (LMICs); 78% of deaths in 2016 from NCDs occurred in LMICs [9]. Even in high income countries, the sections of their population most vulnerable to NCDs are those of low socio-economic status [10]. As with climate change, the economic and social burden of non-communicable diseases hinders the productivity and socio-economic development of countries.

Yet, given the magnitude of the effect that both these challenges pose to health and wellbeing individually, it is concerning that little attention has been given to the combined effect of climate change and NCDs. Compared to the attention given to health outcomes such as mortality, injuries and the spread of vector-borne diseases linked to climate change drives (e.g., extreme weather events, shifting rainfall patterns), the connection between NCDs and climate change has been left largely unexplored to date [8,11,12,13]. Furthermore, most of the research examining the association between NCDs and climate change has been general in scope [11,13,14,15]. The existing literature on the topic of climate change and NCDs can be divided into three streams: (1) articles that broadly describe potential connections between non-communicable diseases and climate change [13,15,16,17]; (2) articles outlining future research needs and priorities to better understand the association between NCDs and climate change [11] and (3) articles that bring attention to policy opportunities (adaptation and GHG mitigation) that confer co-benefits for planetary and human health [13,15,18,19,20,21,22,23]. This literature does not speak to the experiences of individual countries or populations, and so is not conducive to place-specific intervention strategies that consider the vulnerabilities and adaptation needs of populations.

Researchers have proposed that the incidence of NCDs such as cardiovascular diseases, certain cancers, respiratory illnesses, mental-health related illnesses, malnutrition and injuries may be exacerbated by climate change drivers, primarily increases in temperatures and decreases in rainfall; urban air pollution (stratospheric ozone); and extreme weather events [13,15]. Others have proposed that climate change is an additional emerging risk factor to the development of NCDs and the adverse health experiences of persons with NCDs [13]. Moreover, when considering the effects of climate change on the health of populations, consideration must be given to the environmental and institutional contexts (e.g., suitable infrastructure for physical activity, access to health services) within which these drivers are experienced, as well as social and behavioral determinants (e.g., dietary habits; socio-economic status), in order to understand the resultant health outcomes [24].

Furthermore, the relationship between non-communicable diseases and climate change stems from the macro-economic processes that contribute to anthropogenic climate change—industrialization, urbanization and globalization—that also underpin lifestyle habits that have led to the increased prevalence of NCDs [25]. For instance, economic development has contributed to a change in dietary habits, including the quantity and quality of food people consume and thus the nutritional value received from foods. Land use patterns have changed to facilitate the agricultural landscapes needed to provide food for growing populations and meat intensive diets are associated with high methane emissions [2]. Economic development has also changed the way people move from place to place, eschewing healthier modes of transport like walking and cycling in favor of vehicular modes of transport that contribute to carbon emissions. Recognizing these connections is important as it lends credence to compelling arguments that speak to the potential for co-benefits, the redirection and optimization of resource use, and it brings attention to the compounding effect climate change and NCDs may have on population health.

### 1.1. Study Context

The research described in this paper responds to the need for place-specific studies that consider the particular needs of vulnerable populations. It explores attitudes and knowledge of public health professionals involved in combatting the prevalence of non-noncommunicable diseases in Barbados, on the relationship between non-communicable diseases and climate change.

Barbados is the easternmost small island developing state in the Caribbean, with a land area of 430 km^2^ and a population of 277,821 [26]. In the post-independence era, significant strides have been made to improve the health of its population. For instance, the country has reduced the burden of infectious diseases, parasitic diseases and nutritional deficiencies to the point that the epidemiologic profile of the country is more reflective of a developed country [27]. Barbados has made a commitment to provide universal healthcare access to all its population. This commitment has been met with an increase in the quality of health care services across the country made possible with a range of supporting infrastructure to ensure health care is widely accessible [27].

Advancements in health in Barbadians are closely tied to the socio-economic development of the country. However, that has also brought along its own set of challenges, that being the increasing prevalence of chronic lifestyle diseases. Like many Caribbean countries, Barbados suffers with high rates of NCDs and the associated risk factors [27,28,29,30,31]. One quarter of all adults in Barbados have a non-communicable disease (e.g., cardiovascular diseases, diabetes, chronic respiratory diseases, cancers), with rates projected to be one in three by 2025, another one quarter are at risk of developing a non-communicable disease [29]. These diseases have been estimated to account for 83% of all deaths in the country [9]. A national survey conducted in 2007 found that 44% of the population reported having at least three risk factors (e.g., alcohol use, tobacco smoking, overweight/obesity and high blood pressure) for developing a non-communicable disease [29]. Further, a 2012 report showed that when maternal and child health are excluded, 80% of visits to community polyclinics in Barbados were for NCD related issues [31]. These diseases are costly to the country. Direct costs include government expenditure on prevention and management of NCDs, the loss of human lives, the household/individual financial burden of medical care, and private sector and non-health sector expenditure [32,33]. The indirect costs include loss of productivity of the workforce due to absenteeism and presenteeism, decreased human capital when workers can no longer partake in the workforce due to their chronic disease, and loss of income and time lost to caretaking responsibilities at the individual and household level [32,33]. Along with the burden from NCDs, Barbados by virtue of its geographic location, physical characteristics, climatic conditions and development challenges, is vulnerable to a range of climate change stressors; these include sea level rise, increased frequency of drought conditions or heavy rainfall events, and greater intensity of extreme weather events like tropical storms and hurricanes [34].

### 1.2. Objective

To explore the knowledge and attitude of health professionals across multiple scales on (1) the current and future burden of non-communicable diseases in Barbados and (2) possible connections between climate change stressors and non-communicable diseases.

### 1.3. Theoretical Framework

Through a political ecology of health (PEH) lens, this study explores the burden of NCDs and the potential connection between climate stressors and NCDs. PEH builds on theoretical conceptualizations of political ecology to situate issues of health and wellbeing within interconnected political, economic, cultural, social and ecological systems. This framework interrogates how these systems shape health outcomes though the spread of disease and the decision-making opportunities accessible to populations [35,36]. Mayer notes that within political ecology and economy, that recognizing “context” is key to understanding the phenomena under study. The various systems within which health-affecting decisions are made and health outcomes are produced are context-dependent and should be studied as such to best understand and prepare for health outcomes affected by climate change [36].

Political ecology of health also recognizes that while the setting of research may be local scale, multi-scalar analyses are needed to illustrate how local health outcomes are shaped by policies or institutions at various scales [36]. This is apt for the Caribbean, where the successes of local public health policies in countries of the Caribbean Community (CARICOM) have been driven by health priorities set at the regional level through a regional integration process [37]. Furthermore, these regional priorities are linked to the agendas of expert international health organization like the WHO and Pan-American Health Organization (PAHO).

Additionally, the PEH framework assists in explaining how diseases are understood and represented by institutions and how these discourses may align or conflict with local understandings [35]. King notes that these discourses are essential to uncovering inequalities in the distribution of power [35], which in turn has implications for access to resources, opportunities and information (adaptive capacity). While this study does not explore the understandings and perceptions of local populations, data from this research would be beneficial for future comparisons and analyses that incorporate these perspectives.

## 2. Methods

### 2.1. Research Design

The research reported herein is part of a larger study that takes the form of a qualitative case study of Barbados, guided by a political ecology of health framework. Steps from the Vulnerability and Adaptation (VA) assessment guidelines from the World Health Organization, were used to inform the research design for this part of the study. The purpose of the VA guidelines is to provide a framework that can be used to asses current and future burdens of disease given the threat climate change poses to the health of populations [38]. Steps from Stage 1 and 2 of the VA assessment guidelines were implemented in this study: framing and scoping the assessment, assessing current and future health burdens of NCDs and exploring projected health impacts of climate change on NCDs. Stage 3 of the guidelines—managing and monitoring risk—is beyond the scope of the current research.

Following the prescriptions outlined in VA guidelines, the study utilized key informant interviews with public health professionals (*n* = 10) from the community, national and regional levels; some participants operate across multiple levels. Participants were selected from organizations that interact with persons living with non-communicable diseases or persons at risk of developing a non-communicable disease. Their roles include the prevention or management of NCDs, improving health literacy or the provision of technical assistance to organizations involved in these roles (Table 1). Participants were selected for their knowledge of the current burden of NCDs, the policies and strategies that have been implemented to control the burden of NCDs, success and failures to-date, the insight into the consideration given to climate change on NCD agendas and to the possible additional burden of adverse health outcomes due to climate change. Community health professionals were deemed as those who provide health care services directed towards a single community and those operating at the national level provide services for the entire population of the country. Health professionals at the regional level were considered to be those who work with organizations where the organizational structure extends beyond the bounds of Barbados to include neighboring countries in the Caribbean.

The study received ethical clearance from a University of Waterloo Ethics Research Committee (ORE #: 22439).

### 2.2. Participant Recruitment

Key informants for this study were identified through internet searches of directories for Barbadian non-governmental-health organizations, a search for governmental health contacts in the national telephone directory and social media profiles of local health businesses and organizations. Potential participants were sent a formal recruitment letter via email, and the letter described the study’s objectives and methods, issued an invitation for their participation, and outlined the confidentiality measures and procedures for informed consent. Others were contacted by telephone using a prepared script that contained the information in the recruitment letters; interested participants were sent the recruitment letter in a follow-up e-mail. Finally, using the snowball sampling technique, other participants were identified during interviews with key informants. Fifteen people were invited to participate in the study; eleven initially agreed to participate and ten were interviewed. Three attempts were made to interview one individual but we were unable to complete that interview due to schedule conflicts. Four people declined to participate; they offered that while the study seemed important, they could not offer any insight to the topic due to their lack of knowledge of climate change, and identified other participants they believed would be more suited to the study. Recruitment was deemed to be complete based on the inclusion of representatives from all levels of analysis (community, national and regional). Furthermore, we conclude that we had reached data saturation when key informants could offer no new insights, and they all identified potential participants who had already been contacted for the study. We posit that the insight from the key informant that was unable to participate due to scheduling conflict was not entirely lost from the study; the reasoning behind this is that one of the key informants interviewed was responsible for the establishment of the governmental department which that person would have represented and still continues to play a key role in that office.

### 2.3. Data Collection

Interviews with key informants followed a semi-structured format using a standardized interview guide (Appendix A). Participants were allowed leeway in the interviews provided that their discussion remained within the scope of the research objective. The interview questions ranged from descriptive (e.g., role of the interviewee in their organization, research related to climate change they have conducted) to storytelling (e.g., how their work on NCDs has changed overtime and how it fits within the mandate of their organization) to opinion questions (e.g., is there a strong awareness of the links between climate, NCDs and wellbeing among the public and policy makers). Interviews lasted between 35 and 60 min and key informants were interviewed at their place of business. All interviews were audio-recorded for transcription at a later date to ensure accuracy of the accounts.

### 2.4. Analysis

Audio-reordered interviews were transcribed verbatim with permission from informants and thematically coded using the Nvivo12 (QSR International, Melbourne, Australia) qualitative analysis software program. A coding manual grounded by the literature, theoretical framework and interview guide was initially created and applied to two randomly selected interview transcripts by the lead researcher. This coding manual was revised during the initial test application as additional themes emerged from the data. The coding template was re-applied to two randomly selected interview transcripts by the lead researcher and reviewed by a second coder to qualitatively assess the level of agreement on emergent themes. Disagreement on emergent themes were used an as opportunity to further hone the codes, but generally, coders were consistent in the identification of themes from the data.

Other measures used to reduce researcher bias during the research process were introduced during the interview process. The lead researcher ended each interview by restating the main ideas that emerged and sought confirmation from respondents that these ideas were accurate. Respondents were also offered the opportunity to follow-up with the researcher if they wished to add any information or clarify anything they said. This was done to provide an avenue for respondent validation; one participant followed up email to clarify a point they had made during the interview and another followed up to provide sources for statistics they quoted.

## 3. Results

Key informants were asked a range of questions about health challenges in Barbados, of which non-communicable diseases was the priority issue identified. The discussions with participants can be classified under three broad themes: current burden of noncommunicable diseases, future burden of NCDs and potential climate change-NCD connections.

### 3.1. Current Burden on Non-Communicable Diseases in Barbados

*“Well right now I think that the number one problem in Barbados in terms of health issues are chronic non-communicable diseases.”*

**—HP3, Doctor**

When asked what they considered to be the greatest health challenge in Barbados, all but one key informant stated that chronic non-communicable diseases or issues related to those diseases were the major health issues they encounter in their respective professions. Health professionals expressed concern about the high prevalence of these diseases given how largely preventable they can be; complications related to undiagnosed/untreated/poorly managed NCDs; state health care spending necessary to provide free or subsidized care to its citizens; and the economic and social burden to the Barbadian society (Table 2).

#### Issues Associated with the Burden of Non-Communicable Diseases

*“Four chronic diseases were identified as the chronic diseases of importance: cardiovascular disease, cancer, diabetes and chronic lung disease. And these four were identified for essentially two reasons. First of all, they accounted for some 80% of all of the deaths in the Caribbean. And also, they all for the most part, shared the same risk factors for their development … Even though we recognize that there are many, many other chronic diseases.”*

**—HP6, Head of the Board of Directors of the Civil Society Health Non-governmental organization (NGO)**

Respondents were asked which NCDs specifically they considered to be of greatest concern. Responses included various forms of cancers, diabetes mellitus, cardiovascular diseases (e.g., strokes), and metabolic risk factors such as overweight/obesity and hypertension (Table 2). Among these responses, the top two concerns were diabetes and hypertension. Predictably, the diseases reported by health professionals are all on the list of top non-communicable diseases responsible for ill-health in the Caribbean region. Key informants raised another important issue, that of comorbidities. Based on their experiences, medical health professionals noted the difficulty in selecting a single disease of concern as many persons are living with multiple NCDs. They argued they are less concerned about specific NCD diagnoses, but rather the behaviors exhibited by persons and the environments within which they live, that hinder the prevention or management of NCDs. For example, one participant stated:

*“I’m not concerned about one NCD over another, because usually you don’t have one alone. You have diabetes and more likely, not controlled is going to lead to retinopathy or poor circulation so, it progressively leads to other things.”*

**—HP1, Pharmacist**

Concerns were raised about the prevalence of NCDs given their preventable nature and complications arising from NCDs. Non-communicable diseases are largely caused by four behavioral risk factors: insufficient physical activity, unhealthy diets, tobacco use and harmful alcohol use which in turn lead to metabolic risk factors such as overweight/obesity and high blood pressure [39]. Likewise, the same risk factors are important in the management of NCDs. These risk factors are can be attributed to macro-socioeconomic processes such as economic transitions, rapid urbanization, industrialization and the globalization of food markets [13,25]. The behavioral risk factors discussed by participants were physical inactivity and unhealthy diets.

Responses from key informants demonstrated an awareness that the individual health affecting behaviors of Barbadians are influenced by external environments—social, cultural and economic—within which persons live. For instance, it was argued that the sedentary lifestyle of many Barbadians has contributed to increasing incidences of overweight/obesity in the population. One key informant described it as a growing sedentary “culture” among Barbadians, where exercise and movement are no longer a regular part of the daily of life of islanders. This is a major problem as one doctor noted that overweight or obese persons are likely to have comorbidities. Respondents linked physical inactivity among Barbadians to income and social status. For example, one health professional hypothesized that sedentary lifestyles are a symptom of the preoccupation with upward economic mobility among Barbadians. Persons who have managed to achieve a certain level of wealth or economic success can afford material possessions that discourage or distract from the incorporation of physical activity into daily lives (e.g., motor vehicles and various forms of electronics or digital technology). Furthermore, the pursuit of materials possessions—which have become an outward symbol of affluence—takes time away from activities that contribute to healthy lives. This is demonstrated in the quote below:

“We need a change in our culture. We have become too materialistic. Looking at the American culture, the BET lifestyle …I think we need to get back to our old ways of living, living more contented. Park the vehicle, ride more, swim more … We like glitz and glam. I got a better car, I got a better house than you. It’s more about the perception out there.”

**—HP1, Pharmacist**

Respondents also noted that that Barbadians are increasingly turning to more “westernized” diets. This is partially a reference to the growth in the fast-food consumption in the country which participants argued Barbadians are eating because (1) of convenience, (2) it is perceived as inexpensive, (3) of taste and (4) many parents and children view these meals as “rewards” for good behavior or academic accomplishments. Further, participants noted from their interactions with person’s living with NCDs, that Barbadian diets commonly consist of foods considered to be unhealthy (foods high in sugars, starches, fats or “empty calories”), again out of convenience, because of affordability. These reasons for food choices support research that determines socioeconomic status to be an important determinant of health:

“There are socioeconomic impacts with food choices as well. It’s quicker, cheaper to purchase, starchy foods. Just to get by your day, essentially feed your kids foods like hot dogs, that type of thing, but ultimately, it’s a huge impact on the health of the greater population.”

**—HP10, Retired nurse**

The poor management of non-communicable diseases and their risk factors by Barbadians, leads to avoidable, debilitating complications or disabilities which can affect quality of life and the ability to be productive citizens; in some cases, the outcome is death. Key informants suggested that many of these complications arise because there are people living with undiagnosed chronic diseases that have never sought screening, even though opportunities are regularly offered free of cost to Barbadians. They offered that many Barbadians fail to take advantage of screening opportunities as they experience no symptoms until their conditions, having been left untreated, progresses to a dangerous stage. For example, one respondent noted:

“Diabetes is one of the biggest silent killers in Barbados. Diabetes is also preventable through good diet and physical activity. Diabetes also has the highest disability factor from problems with microcirculation. It is the main cause of blindness, the main cause of kidney disease, the main cause of amputation.”

**—HP7, Pan American Health Organization (PAHO) representative**

Another reason for these complications is that there are many persons who are non-compliant with their medication care plans, even though the cost of medication is not prohibitive, again because they do not experience any symptoms. One doctor stated:

“I think that what makes hypertension a little more dangerous is because people don’t feel hypertension so it’s very easy for them to take the same free medication and just not use it. Sometimes you will forget to take it, and it’s not like diabetes where you might start to feel sluggish, or you might start to feel ill because you’ve not taken your medication. With high blood pressure you could be going along with pre-stroke high blood pressures and not feel it, you won’t get headache, you won’t get any problems until it’s too late.”

**—HP4, Doctor**

Interestingly, the doctors proposed that another reason for the extreme complications arising from poorly managed NCDs is a lack of confidence in health care providers. They were specifically referring to the distrust patients have for doctors as it relates to a stigma in Barbados around amputations. They observed that Barbadians diagnosed with diabetes have a fear that if they attend a doctor, their limbs will be amputated:

“That stigma has been a big problem and has also often impeded the relationship between doctors and patients because there are some patients who already come with the mindset that this person wants to take my foot … I’m someone who is interested in general surgery, and from what I’ve seen there are far too many people that end up with amputations. And it’s not because they come to the hospital and we’re surgeons and we’re trying to cut off their foot, it’s because they have come to the point where, to save their life, we have to take their foot.”

**—HP3, Doctor**

### 3.2. Future Burden of NCDs in Barbados

*“When it comes to reducing the burden of NCDs, unfortunately very few of the Caribbean countries, Barbados included, have managed to reduce the burden of NCDs. In fact, the burden of NCDs is increasing and not decreasing …The burden has been increasing exponentially and the burden is now at a stage where it’s crippling. If you look at Queen Elizabeth Hospital, if you look at the health centers, the majority of their patient load is for NCDs.”*

**—HP7, PAHO representative**

All key informants agreed that the burden of chronic non-communicable diseases has been increasing in Barbados. Growing incidence of NCDs continues despite the varied responses, which include the establishment of a dedicated department within the government to address this problem (National Non-communicable Disease Commission); strategic health plans to direct action on NCDs, sustained education programs to disseminate information to citizens; the introduction of policies and legislations to address risk factors; inter-sectoral cooperation; multi-governmental collaborations at the regional level; and the active involvement of civil society. Key informants posited that efforts to reduce the burden of these diseases are hindered by economic, social and cultural and factors as well as individual behaviors (Table 3). Preoccupation with wealth and economic prosperity on the part of both individuals and businesses within the private sector conflict with good physical wellbeing. On the other hand, there are many Barbadians with limited income, which is known to be linked to poorer health. While Barbados does provide access to healthcare free of cost or heavily subsidized, participants noted the healthcare system is overburdened, due in part to the high prevalence of NCDs, and so there is concern about the capability to maintain access to these services at the level to which Barbadians have become accustomed. Already, the leadership of Barbados has proposed fiscal austerity measures which would cut some social protection programs in place. Interestingly, a number of respondents also noted that because health care is fairly accessible, there is much greater focus on the treatment of chronic diseases rather than prevention. Some participants also noted that despite educational health campaigns, there remains pervasive misconceptions about how Barbadians can improve their health, leading them to make poor lifestyle decisions.

Furthermore, some participants reported that along with an increasing prevalence of NCDs, they are observing is a greater number of younger people being diagnosed with non-communicable diseases. They made these comments based on their own experiences and from discussions with colleagues who have been in the profession for a longer time. For example:

“I remember one of the discussions we had was about the kind of patient that’s coming in, and we’re noticing the chronic illnesses a lot earlier. So, it’s not that you are seeing people that are 50 and up coming in for the first time with high blood pressure and diabetes; you see it in their 30s and 40s and sometimes even in the younger people. There’s a lot, more pediatric cases of diabetes type 2. You’re seeing more of the pediatric onset of what we thought was an adult illness.”

**—HP4, Doctor**

### 3.3. Climate Change—Non-Communicable Diseases Connections

#### 3.3.1. Disparities in Knowledge of Climate Change and Health Impacts

*“You know there’s not really much consideration given to the effect of the climate on NCDs, not at the places I have worked.”*

**—HP4, Doctor**

There was a disparity in the knowledge of climate change impacts on health possessed by the health professionals who participated in this study. The health professionals who operate at a regional level had a greater knowledge of the climate change impacts on health or were more able to postulate on the potential connections between climate stresses and non-communicable diseases. Health professionals operating at solely the community level were generally unaware of these connections (Table 4). The representative from the PAHO was the most knowledgeable on the topic of climate stressors and chronic non-communicable diseases, and was able to discuss at length a range of potential climate change health impacts. Similarly, the health professional representing the civil-society health NGO, noted that while they were not overly familiar with the multitude of ways climate stresses could affect the prevalence of NCDs or the adverse experiences of people living with NCDs, this was an issue that the organization has considered for future work. This participant was still able to offer some ideas on the potential connections between climate change impacts and non-communicable diseases as is described in detail below. Participants who work with organizations that operate at the national and community level, were also less familiar with the connection between climate stresses and NCDs but were nonetheless able to offer some insight based on their expertise on NCDs and their limited knowledge of climate change. Health professionals from community level organizations were unable to offer any information on connections between climate stresses and NCDs. The participants who declined to participate in this study indicated that they were unaware of a connection between climate change and NCDs. Furthermore, responses from these health professionals about the specifics of climate change impacts in Barbados, suggest that they don’t possess enough knowledge about climate change to be able to speak to any potential connections to chronic non-communicable diseases. This lack of knowledge is indicative of an overall lack of attention on climate change on the parts of the organizations where they are employed and greater lack of attention from the Barbadian health system as a whole. In fact, some of these participants indicated that this was not a topic to which they had given much, or any, thought. We cannot state that knowledge of climate change and health is correlated to the level of the organization where respondents are employed, nor do we wish to as this was not tested. However, these results do raise the possibility that the knowledge possessed by health professionals is linked to the information available to certain organizations and warrants further investigation to test this hypothesis.

#### 3.3.2. Overlap between Social Determinants of Health and Determinants of Vulnerability

*“We speak of chronic disease being lifestyle driven, but if you go further upstream, they are really first driven by what we call the social determinants: where you live, where you play, whether you are empowered as a person, your stress levels and so on … These adverse social determinants are far more common in social class four. This is the social class more likely to get chronic diseases, and it’s also the class that is most significantly impacted by hurricanes.”*

**—HP6, Head of Board of Directors of civil society health NGO**

One key informant commented on the commonalities between social determinants of health that contribute to the prevalence of chronic non-communicable diseases and the factors that affect the vulnerability of populations to the impacts of climate change. These determinants included income, education, occupation, social support, exposure to environmental hazards and access to health services/resources. Similarly, these social determinants are factors that affect the exposure, sensitivity and adaptive capacity of populations to climate change. Further, inequalities in the distribution of chronic non-communicable diseases among populations are likely to mirror the vulnerable populations disproportionately impacted by climate change.

#### 3.3.3. Climate Change Impacts Could Affect NCD Prevention and Management

*“We’re telling people to eat fresh fruits and vegetables but then how many fresh fruits and vegetables can you find on the market? And how much is available across the island or is it in specific areas? So, availability is limited, and affordability is even more limited.”*

**—HP7, PAHO representative**

Key informants proposed that climate stresses could hinder the prevention and management of NCDs through the pathway of modifiable behavioral risk factors like unhealthy diets and physical inactivity.

##### Food Security/Sustainability

Poor nutrition is one of the key risk factors to the development of NCDs, where the calories and types of food consumed greatly affect the likelihood of developing metabolic risk factors like obesity/overweight and hypertension. In this way, key informants proposed a link between the impacts of climate change on food security and the prevention of NCDs through accessibility to high quality, affordable food. The examples given by key informants were related to the agricultural and fisheries sectors in Barbados. For example, one respondent noted:

“We already have a difficulty in getting affordable fresh fruits and vegetables on the market. Now if climate change effects, or weather effects are going to continue to damage our produce, it’s going to get even worse.”

**—HP7, PAHO representative**

As it relates to the fishing industry, they said:

“If we are going to have a further increase in our sea water temperatures, the whole issue of the sustainability of fishing. We saw a few years ago the whole issue of the Sargassum weed, and how that affected the whole food chain with regards to the fishing industry. Fishing has already declined as an economically viable industry in the region, so we’re relying on imported products, imported frozen fish from other sides of the world with the additional processing.”

**—HP7, PAHO representative**

Similarly, another respondent noted that already Barbadians perceived “healthy foods” like fruits and vegetables as expensive, which affects their food choices; opt to buy “cheaper”, processed foods that have a lower nutritional value. Given these behaviors in what could be considered ‘normal’ conditions, key informants proposed climate change stressors like heavy rainfall, increasing sea surface temperatures and natural disasters, could affect the availability and affordability of local foods on a more frequent basis. This would likely further negatively influence the food choices available to Barbadians. This is not an unlikely scenario for Barbados; in a local newspaper in 2017, the Chief Executive Officer of the Barbados Agricultural Society noted that heavy rainfalls had impeded local farmers’ ability to prepare their fields for harvest, which resulted in a shortage of local vegetables and a price hike for consumers [40].

Another participant noted that changing environmental conditions could detract from the attractiveness of occupations like farming and fishing for income generation. This would then have a trickle-down effect on the quantity of local foods available for market and consumption. With less local foods available, Barbados will be more reliant on imported food to meet the needs of citizens.

*“People are staying away from the outdoors in terms of farming and doing stuff that could probably produce foods that would be more, what we would call healthy to use.”*

**—HP8, Nutritionist**

##### Physical Inactivity

Some key informants proposed that changes in climate or changes to the built environment related to climate could influence Barbadian attitudes toward physical activity. The climatic changes to which key informants were referring were increases in ambient temperatures (hotter days), unpredictable weather patterns (heavy rainfall) and damage to the built environment from extreme weather events. For example, one participant stated:

“We don’t have a lot of active lifestyles because everyone wants to be in their car with the AC on, because they say it’s hot.”

**—HP8, Nutritionist**

Another participant discussed the challenges of cultivating routines that incorporate physical activity into people’s daily lives because of the unpredictability of the weather or disruptions to the built environment that hinder physical activities. The cumulative effect of these environmental conditions is increasingly negative attitudes towards physically active lifestyles, adding to the growing sedentary culture of Barbados. This is demonstrated in the following quote:

“We had practically a whole month where it rained every day. Totally unusual for Barbados. You couldn’t tell your children to walk to school because you didn’t know if in the afternoon it will rain. I mean yesterday, case in point. It was a beautiful day in the beginning of the day. Hot, nice, sunny. By lunch time, torrential rain till the night.”

**—HP7, PAHO representative**

##### Vulnerable Populations

As it relates to the management and treatment of chronic diseases, two key informants noted that people living with chronic non-communicable diseases constitute a vulnerable population to climate change. This vulnerability is due to the additional exposures and sensitivities this population face related to their need for access to regular, on-going health services and resources. This access may be disrupted during and after the passage of extreme weather events. For instance, one health professional said:

“We already don’t have enough dialysis facilities for the public to accommodate the number of persons that are going into kidney failure. Imagine, if we do have a natural disaster that affects these facilities, those people honestly and unfortunately they will probably die.”

**—HP5, Doctor**

Similarly, another noted:

“One of the consequences of weather events is people being cut off. People might have a week’s supply of medication and if the communication and the connectivity with the town or with the nearest health center is not there, how do they get access to their medications?”

**—HP7, PAHO representative**

#### 3.3.4. Physiological and Psychosocial Impacts of Climate Change

Other climate change—NCD connections discussed by respondents were direct physiological effects of climatic stressors. These were the potential for heat strokes brought on by heat waves, poor quality air from increased vehicular use and the impacts on chronic lung diseases as reflected in the quotes below:

“There is the increased use of vehicles which then impacts physical activity, and there are also the carbon emissions from these vehicles that also has an impact then on the chronic lung diseases.”

**—HP6, Head of Board of Directors of civil society health NGO**

Finally, two key informants expressed concern that climate change impacts would affect the psychosocial wellbeing of Barbadians. They posited that this could be related to social effects of food insecurity and water insecurity, and the immediate and long-term effects of (repeated) exposure to extreme weather events. This is demonstrated in the quotes below:

“The stress of having a weather event. Think about how that affects your blood pressure and how that affects your blood glucose and how that affects all your complications arising from the NCD which you are living with.”

**—HP7, PAHO representative**

## 4. Discussion

This study adds to a small body of knowledge on the effects of climate change on health in the Caribbean, and to the limited pool of research on the NCD prevention and climate change. Previous studies have been successful in directing attention to issue of the relationship between NCDs and climate change, however, the application of findings from those studies to local health interventions is limited by the broad scope of the research. Theory-driven empirical research has demonstrated how local health outcomes are produced within inter-connected social, cultural, political and economic landscapes that differ from place to place. On the other hand, given the case study approach utilized in this research, though the data is richer, it is also highly contextual and therefore may not reflect the full complexities of the effects of climate change on NCDs in other locations. This does not discount the utility of this study as there are lessons that can be applied to places with similar contexts. For instance, small island states around the world have been recognized to share similar physical characteristics, developmental challenges and likely similar vulnerabilities to climate change [41]. A nuanced understanding of the situation in Barbados is beneficial as a starting point for countries or communities assessing their own vulnerabilities to climate change in order to implement health interventions or climate change policies to mitigate the impacts of climate change. Even more useful are lessons from the research design and the methodology implemented to collect data.

The Vulnerability and Adaption assessment guidelines from the WHO provide one mechanism for assessing health impacts of climate change at the national or subnational level [38]. The methodology is adaptable based on the needs of users. Usually, these assessments would be implemented through a ministerial body such as a Ministry of Health or team responsible for climate change planning within the government. In such cases, access to funding, human capital to carry out the assessment and expert knowledge from stakeholders would be more widely available, allowing for greater scope. This study was not intended to be a full assessment. Even still, the WHO assessment guidelines were useful in guiding methodology with regard to the selection of the geographic region, health outcomes of interest, participant selection, data collection methods and tools. While the application of VA assessment guidelines in this study is not its first use in the Caribbean [42], it is the first to focus on non-communicable diseases, providing support for the utility of the guidelines to research a variety of health outcomes. A limitation of this study is the involvement of only health professionals to understand the current and future vulnerabilities to climate change. These professionals were selected for the insight they could provide on the state of knowledge of climate change within the local health sector, and the priorities on local health agendas. However, there are other stakeholders who could provide insights to this issue. Future studies could explore attitudes and perspectives of average citizens as they are the ones that will be directly impacted by climate change or involve stakeholders from outside the health sector.

Health professionals believe the burden of chronic diseases in Barbados will continue to grow to the detriment of individuals and the wider society. They base this on the current prevalence of chronic diseases in Barbados, the attitudes and behaviors of persons living with NCDs, the lifestyles of Barbadians, and the environments within which the live. Respondents drew attention to the effect of economic, social, and cultural factors on the health outcomes of Barbadians. Major findings from a national survey on NCDs and their risk factors, outlines the extent of the NCD crisis in Barbados and lends support to position of health professionals. Two in three adults are overweight; one in three are hypertensive; one in five have diabetes and at least one in three of persons receiving treatment for hypertension or diabetes had sub-optimal control of their disease [43]. This is compared to infectious diseases and malnutrition which have been on the decline due to successful interventions and improved social conditions [29]. Even with a policy landscape that should be supportive of healthier day-to-day choices from the Barbadian population, the NCD crisis continues.

To reduce mortality from NCDs in Barbados by 25% by 2025—one of the targets set by the United Nations [44]—greater efforts are needed given the lack of success thus far, despite the widespread attention this issue has attracted. The social, cultural and economic landscape in Barbados continues to foster an environment where the incidence and prevalence of NCDs increases unabated. This should be even more concerning to public health leaders and decision makes, given the rising threat of climate change which will likely amplify the future burden of NCDs, through some of these existing factors.

It is troubling that plans to tackle the NCD crisis in Barbados and improve health outcomes have been discussed under the assumption of a stable climate. This is evidenced by the lack of concern from health professionals that participated in this study about the future burden of NCDs in the context of a changing climate as. This is largely because of inadequate knowledge about climate change among the majority of the health experts interviewed. Those knowledgeable about potential connections, noted the difficulty that climate change would pose to the prevention and management of NCDs, given the impacts of climate stressors to food security, the built environment, as well as the physiological and psychosocial impacts. Health systems cannot effectively plan interventions for NCDs without considering future climate stressors and the effect they will have on the strategies to prevent and manage these diseases. For instance, interventions designed to encourage more outdoor physical activity by improving the walkability of a neighborhood, that neglect to consider climate stressors like increased temperatures or more regular heavy rainfall events, are destined to fail. For such an intervention to be successful, it needs to consider the impacts that climate change will have on the built environment and the attitudes or behaviors of the people for whom the interventions have been designed. Another example would be the introduction of educational campaigns that promote the benefits of certain types of food, without thinking about how climatic conditions would affect accessibility to those foods. It may necessitate the involvement of government and political leadership to make those foods more accessible through welfare programs, subsidies or other market-control measures that ensure access to all members of society, irrespective of their socio-economic status.

Lack of awareness of climate change from health professionals interviewed is symptomatic of the low priority ascribed to climate change on the national health agenda. Even more telling, is that the health professionals most knowledgably about the relationship between NCDs and climate change, are those who work with regionally operating agencies. This is suggestive of a top-down flow of information, with a clear influence of international/regional bodies like the World Health Organization and the Pan-American Health Organization. This hints at the importance of international agendas in shaping local health priorities and agenda setting. Yet, it seems that the knowledge and awareness of the climate risks to NCDs within these regional/international bodies has not reached the magnitude needed to influence policy development at the national level. However, the flow of information and influence on agenda setting does not need to unidirectional from the top-down, as has been demonstrated by a display of Caribbean leadership in setting a global NCD agenda. A meeting of CARICOM Heads of States to discuss the challenge of NCDs in their countries and their commitment to action in the form of the Port-of-Spain Declaration, was the first high level meeting of its kind in the world [45]. Furthermore, leaders from the Caribbean played a big role in advocating for the United Nation High Level Meeting on NCDs from which the United Nation Political Declaration on NCD prevention and control emerged [45]. Since then, there have been other global efforts to motivate and help countries to prevent and control NCDs such as the WHO Global Action Plan for NCD prevention [44] and the 2030 Agenda for Sustainable Development Goals [46]. Currently, there is inadequate availability of climate change information to local stakeholders to motivate political leadership to advance a policy response to climate change risks to health. This is an opportunity for research to fill these gaps in knowledge through the involvement of a variety of key stakeholders in collaboration with academic institutions. Giving stakeholders the opportunity to participate in the knowledge creation process, could encourage them to take ownership of that knowledge and use it to advance goals that protect and improve the health and wellbeing of citizens. These stakeholders, equipped with enough information, could be influential in driving national, regional and global policy momentum from the bottom-up. The stakeholder involved should represent a multisectoral approach to addressing this issue, given the study’s findings that climate change stressors will intersect with NCDs in various ways that would require the involvement of sectors beyond health. This might include the representatives from the fisheries and agricultures sector, health sector, private sector, disaster management agencies, town and country planners, school boards, sports organizations, faith-based organizations, community groups, parents, teachers and members of the general public who stand to be directly impacted.

## 5. Conclusions

This study found that health professionals have deemed non-communicable diseases to be a major health problem in Barbados, currently and into the future. Comparatively, only health professionals working with institutions that operate at the regional level displayed awareness of the risk climate change poses to NCD prevalence and outcomes. There is a need for the dissemination of information on the health risks of climate change to stakeholders in the health sector so as to increase the attention given to climate change, strengthen advocacy for climate change adaptation planning within health systems and encourage political leadership in Barbados to give priority to climate change adaptation planning on development agendas. Furthermore, given the connections between NCDs and climate change identified in this study, we reiterate the need for a multisectoral response to the dual burden of NCDs and climate change, recognizing that health and wellbeing are affected by factors that exist beyond the boundaries of solely the health sector.

## Figures and Tables

**Table 1:** Description of key informants.

Participant ID	Profession	Level of Operation of Organization	Description of Role
Health professional (HP) 1	Pharmacist	Community	Management of NCDs
HP 2	Pharmacist	Community	Management of NCDs
HP 3	Doctor	Community, national	Management and prevention of NCDS
HP 4	Doctor	Community	Management of NCDs
HP 5	Doctor	Community, national	Management and prevention of NCDS
HP 6	Head of Board of Directors of civil society health non-governmental organization NGO	National, regional	Prevention of NCDs, Health Literacy
HP 7	Representative from Pan American Health Organization (PAHO)	Community, national, regional	Technical assistance, Management and prevention of NCDS
HP 8	Nutritionist	Community, national	Management and prevention of NCDS
HP 9	Fitness instructor/personal trainer	Community	Prevention of NCDs
HP 10	Retired nurse	Community	Management and Prevention of NCDS, Health Literacy

**Table 2:** Key informant views on health challenges in Barbados.

Health Challenges	Specific Diseases/Issues	# of Participants Mentioning *n*/10 (%)	# of Mentions
Chronic non-communicable diseases	Diabetes	10 (100)	25
	Cardiovascular diseases	8 (80)	8
	Other NCDs (cancers, lung diseases, etc.)	2 (20)	3
	Behavioral risk factors (unhealthy diets, physical inactivity)	8 (80)	11
	Metabolic risk factors (overweight/obesity, hypertension)	10 (100)	20
	Complications from undiagnosed/untreated/poorly managed diseases (e.g., amputations “diabetic foot”, kidney diseases, blindness)	5 (50)	8
	Cost to state and society	8 (80)	11
	Mental health illnesses	4 (40)	5
Infectious diseases	Zika, Chikungunya, Ebola	1 (10)	2
Extreme weather events/natural disasters	Post-disaster access to care	2 (20)	3

**Table 3:** Factors hindering the reduction in the burden of healthcare in Barbados.

Determinants	Factors
Economic	Overburdened healthcare system Limited resources (financial and human) for response to the NCD crisis Competing goals from the private sector Fiscal austerity measures
Social	Income Access to health services Health Education
Cultural	Shift from traditional to “westernized” diets Different priorities for a “good life”
Individual behaviors	Noncompliance with medication regimes Health care avoidance

**Table 4:** Key informant knowledge of climate change and health impacts.

Profession	Level of Organization	Climate Change & Health Impacts	Connection between Climate Stresses & NCDS
Pharmacist	Community	No	No
Pharmacist	Community	No	No
Doctor	Community, national	Yes	Yes
Doctor	Community	Yes	No
Doctor	Community, national	Yes	Yes (with prompting)
Board of Director of health NGO	National, regional	Yes	Yes
Representative from PAHO	National, regional	Yes	Yes
Fitness instructor/personal trainer	Community	Yes	No
Retired nurse	Community	No	No
Nutritionist	Community, national	Yes	Yes

## Appendix A

**Table A1:** Sample Interview Questions from semi-structured Interview Guide with Key informants/experts.

Purpose: To gather insight from key health stakeholders about their perceptions and experiences with NCDs (current and future concerns); To provide an insight into the awareness and consideration of linkages between climate change and NCDs		
Construct	Primary Question	Secondary Question/Probe
**Context**	What is the full name of your organisation? Can you tell me about the formation/history of your organisation? Can you tell me about yourself? What are [has been] some of the biggest health challenges you have observed over the years?	What is its mandate? Has it changed over time? Can you describe your role in your role in your organisation? How long? What brought you to this position? How have these changed over time?Which ones have changed?
**Perception and experiences of climate change, health and NCDs**	How has the burden of NCDs on the health care system changed over time? Are they any specific NCDs that you are concerned about?	What do you think is accounting for these changes? Why/Why not?
	Do you think climate change is a problem? Are you aware of health impacts of climate change? Are you aware of any linkages between NCDs and climate change?	When you hear climate change impacts in Barbados, what comes to mind? What are they?
	Do you that climate change would affect NCD sufferers and persons at risk of developing NCDs?	
**General/Concluding thoughts**	Is there anything else you would like to add that we have not already discussed? Is there anyone else you think we should talk to NCDs or climate change and health?	

## References

[1] World Health Organization (WHO) Ten Threats to Global Health in 2019. https://www.who.int/emergencies/ten-threats-to-global-health-in-2019.

[2] Climate Change 2014: Synthesis Report. Contribution of Working Groups I, II and III to the Fifth Assessment Report of the Intergovernmental Panel on Climate Change. https://www.ipcc.ch/report/ar5/syr/.

[3] Intergovernmental Panel on Climate Change (IPCC) Climate Change 2007: Synthesis Report. Contribution of Working Groups I, II and III to the Fourth Assessment Report of the Intergovernmental Panel on Climate Change. https://www.ipcc.ch/report/ar4/syr/.

[4] Costello A., Abbas M., Allen A., Ball S., Bell S., Bellamy R., Friel S., Groce N., Johnson A., Kett M. (2009). Managing the health effects of climate change: And University College London Institute for Global Health Commission. Lancet.

[5] McMichael A.J., Woodruff R.E., Hales S. (2006). Climate change and human health: Present and future risks. Lancet.

[6] Smith K.R., Woodward A., Campbell-Lendrum D., Chadee D.D., Honda Y., Liu Q., Olwoch J.M., Revich B., Sauerborn R. (2014). Human health: Impacts, adaptation, and co-benefits. Climate Change 2014: Impacts, Adaptation, and Vulnerability. Part A: Global and Sectoral Aspects. Contribution of Working Group II to the Fifth Assessment Report of the Intergovernmental Panel on Climate Change.

[7] Watts N., Adger W.N., Agnolucci P., Blackstock J., Byass P., Cai W., Chaytor S., Colbourn T., Collins M., Cooper A. (2015). Health and climate change: Policy responses to protect public health. Lancet.

[8] Quantitative Risk Assessment of the Effects of Climate Change on Selected Causes of Death, 2030s and 2050s. https://apps.who.int/iris/bitstream/handle/10665/134014/9789241507691_eng.pdf.

[9] Non-Communicable Diseases Country Profiles 2018. https://www.who.int/nmh/publications/ncd-profiles-2018/en/.

[10] Bennett J.E., Stevens G.A., Mathers C.D., Bonita R., Rehm J., Kruk M.E., Riley L.M., Dain K., Kengne A.P., Chalkidou K. (2018). NCD Countdown 2030: Worldwide trends in non-communicable disease mortality and progress towards Sustainable Development Goal target 3.4. Lancet.

[11] Colagiuri R., Boylan S., Morrice E. (2015). Research Priorities for NCD Prevention and Climate Change: An International Delphi Survey. Int. J. Environ. Res. Public Health.

[12] Haines A., Kovats R.S., Campbell-Lendrum D., Corvalan C. (2006). Climate change and human health: Impacts, vulnerability and public health. Public Health.

[13] Kjellstrom T., Butler A., Lucas J., Bonita R. (2010). Public health impact of global heating due to climate change: Potential effects on chronic non-communicable diseases. Int. J. Public Health.

[14] Bauchner H., Fontanarosa P. (2014). Climate change: A continuing threat to the health of the world’s population. JAMA.

[15] Friel S., Bowen K., Campbell-Lendrum D., Frumkin H., McMichael A.J., Rasanathan K. (2011). Climate Change, Noncommunicable Diseases, and Development: The Relationships and Common Policy Opportunities. Annu. Rev. Public Health.

[16] Acharibasam J.W., Anuga S.W. (2018). Psychological distance of climate change and mental health risks assessment of smallholder farmers in Northern Ghana: Is habituation a threat to climate change?. Clim. Risk Manag..

[17] Kjellstrom T., Kovats R.S., Lloyd S.J., Holt T., Tol R.S. (2009). The direct impact of climate change on regional labor productivity. Arch. Environ. Occup. Health.

[18] Beaglehole R., Bonita R., Alleyne G., Horton R., Li L., Lincoln P., Mbanya J.C., Mckee M., Moodie R., Nishtar S. (2011). UN high-level meeting on non-communicable diseases: Addressing four questions. Lancet.

[19] Friel S. (2010). Climate change, food insecurity and chronic diseases: Sustainable and healthy policy opportunities for Australia. N. S. W. Public Health Bull..

[20] Islam S.M.S., Purnat T.D., Phuong N.T.A., Mwingira U., Schacht K., Fröschl G. (2014). Non-Communicable Diseases (NCDs) in developing countries: A symposium report. Glob. Health.

[21] Mash R. (2010). Chronic diseases, climate change and complexity: The hidden connections. S. Afr. Fam. Pract..

[22] McMichael A.J., Powles J.W., Butler C.D., Uauy R. (2007). Food, livestock production, energy, climate change, and health. Lancet.

[23] Song G., Li M., Fullana-i-Palmer P., Williamson D., Wang Y. (2017). Dietary changes to mitigate climate change and benefit public health in China. Sci. Total Environ..

[24] The Impacts of Climate Change on Human Health in the United States: A Scientific Assessment.

[25] World Health Organisation (WHO) (2005). Preventing Chronic Diseases —A Vital Investment.

[26] Barbados Statistical Services (BSS) Barbados Population and Housing Census 2010. http://www.barstats.gov.bb/files/documents/PHC_2010_Census_Volume_1.pdf.

[27] The Health of the Nation is the Wealth of the Nation: Barbados Strategic Plan for Health 2002–2012. apps.who.int/medicinedocs/documents/s18831en/s18831en.pdf.

[28] Pan American Health Organization (PAHO) (2012). Health in the Americas, 2012—Regional Outlook and Country Profiles.

[29] Pan American Health Organization (PAHO) (2012). Health in the Americas 2012 Edition: Country Volume—Barbados.

[30] Chief Medical Officer’s Report, 2010–2012. https://www.barbadosparliament.com/uploads/sittings/attachments/05530f55cc1d0cd01198dbc26b76209b.pdf.

[31] Barbados Strategic Health Plan for the Prevention and Control of Non-Communicable Diseases 2015–2019. https://extranet.who.int/nutrition/gina/sites/default/files/BRB_2015_SPNCDs_0.pdf.

[32] The Global Economic Burden of Non-Communicable Diseases. http://www3.weforum.org/docs/WEF_Harvard_HE_GlobalEconomicBurdenNonCommunicableDiseases_2011.pdf.

[33] Kankeu H.T., Saksena P., Xu K., Evans D.B. (2013). The financial burden from non-communicable diseases in low-and middle-income countries: A literature review. Health Res. Policy Syst..

[34] Review of Health Effects of Climate Variability and Climate Change in Caribbean. http://dms.caribbeanclimate.bz/M-Files/openfile.aspx?objtype=0&docid=2707.

[35] King B. (2010). Political ecologies of health. Prog. Hum. Geogr..

[36] Mayer J. (1996). The political ecology of disease as one new focus for medical geography. Prog. Hum. Geogr..

[37] Theodore-Gandi B., Barclay G. (2008). Protecting and improving the health of the Caribbean people. Am. J. Public Health.

[38] Protecting Health from Climate Change: Vulnerability and Adaptation. http://wwww.who.int/globalchange/resources/adaptationresources/en/.

[39] Global Status Report on Noncommunicable Diseases 2014 (No. WHO/NMH/NVI/15.1). https://apps.who.int/iris/bitstream/handle/10665/148114/9789241564854_eng.pdf.

[40] Vegetables ‘Price Hike’. https://www.nationnews.com/nationnews/news/104225/vegetables-price-hike.

[41] Agenda 21: Programme of action for sustainable development; Rio Declaration on Environment and Development; Statement of Forest Principles: The final text of agreements negotiated by governments at the United Nations Conference on Environment and Development (UNCED), 3–14 June 1992, Rio de Janeiro, Brazil. http://wedocs.unep.org/handle/20.500.11822/2086?show=full.

[42] Verret M., Berry P., Fook T., Lal A. (2018). Assessment of Climate Change and Health Vulnerability and Adaptation in Dominica. PubMed.

[43] The Barbados Health of the Nation Survey: Core Findings. http://www.archive.healthycaribbean.org/newsletters/aug-2015/CDRC_HealthOfTheNationSurvey.pdf.

[44] Global Action Plan for the Prevention and Control of Non-Communicable Diseases 2013–2020. https://apps.who.int/iris/bitstream/handle/10665/94384/9789244506233_rus.pdf.

[45] Health Caribbean Coalition (HCC) Strategic Plan 2017—2021: Enabling Caribbean Civil Society’s Contribution to National, Regional and Global Action for NCD Prevention and Control. https://www.healthycaribbean.org/wp-content/uploads/2017/02/HCC-Strategic-Plan_WEB.pdf.

[46] World Health Statistics 2016: Monitoring Health for the SDGs Sustainable Development Goals. https://www.who.int/gho/publications/world_health_statistics/2016/en/.

